# Central nervous system vasculopathy and Seckel syndrome: case illustration and systematic review

**DOI:** 10.1007/s00381-021-05284-8

**Published:** 2021-08-03

**Authors:** Osama Khojah, Saeed Alamoudi, Nouf Aldawsari, Mohammed Babgi, Ahmed Lary

**Affiliations:** 1 College of Medicine, King Saud Bin Abdulaziz University for Health Sciences, Jeddah, Saudi Arabia; 2 King Abdullah International Medical Research Center, Jeddah, Saudi Arabia; 3 King Abdulaziz Medical City, National Guard Health Affairs, Jeddah, Saudi Arabia; 4 Division of Neurosurgery, King Abdulaziz University Hospital, Jeddah, Saudi Arabia

**Keywords:** Bird-headed dwarfism, Cerebrovascular disorders, Intracranial aneurysm, Microcephalic osteodysplastic primordial dwarfism, Type II, Moyamoya disease, Seckel syndrome

## Abstract

**Purpose:**

To systematically review reported cases of Seckel syndrome (SS) and point out cases associated with central nervous system (CNS) vasculopathy and provide a summary of their clinical presentation, management, and outcomes including our illustrative case.

**Methods:**

We conducted a search on the MEDLINE, PubMed, Google Scholar, and Cochrane databases using the keywords “Seckel + syndrome.” We identified 127 related articles reporting 252 cases of SS apart from our case. Moreover, we searched for SS cases with CNS vasculopathies from the same databases. We identified 7 related articles reporting 7 cases of CNS vasculopathies in SS patients.

**Results:**

The overall rate of CNS vasculopathy in SS patients is 3.16% (n = 8/253), where moyamoya disease (MMD) accounted for 1.97%. The mean age is 13.5 years (6–19 years), with equal gender distribution (M:F, 1:1). The most common presenting symptoms were headache and seizure followed by weakness or coma. Aneurysms were mostly located in the basilar artery, middle cerebral artery, and internal carotid artery, respectively. Regardless of the management approach, 50% of the cases sustained mild-moderate neurological deficit, 37.5% have died, and 12.5% sustained no deficit.

**Conclusion:**

A high index of suspicion should be maintained in (SS) patients, and MMD should be part of the differential diagnosis. Prevalence of CNS vasculopathy in SS is 3.16% with a much higher prevalence of MMD compared to the general population. Screening for cerebral vasculopathy in SS is justifiable especially in centers that have good resources. Further data are still needed to identify the most appropriate management plan in these cases.

**Supplementary information:**

The online version contains supplementary material available at 10.1007/s00381-021-05284-8.

## Introduction

Microcephalic primordial dwarfism (MPD) is a rare form of intrauterine growth retardation (IUGR), which leads to postnatal dwarfism. MPD is a group of disorders that includes Seckel syndrome (SS), microcephalic osteodysplastic primordial dwarfism (MOPD), and Meier-Gorlin syndrome which occur due to embryonic abnormalities [1, 2]. SS (or bird-like dwarfism) is an autosomal recessive microcephalic primordial dwarfism, affecting almost 1 out of 10,000 children with no gender predilection [3]. Few reports of SS have linked the disease with multiple central nervous system (CNS) pathologies such as intracranial aneurysms or severe hypertension resulting in cerebrovascular accidents, agenesis of the corpus callosum, cortical dysplasia, and moyamoya disease (MMD) [4–7]. We report a case of SS that was complicated with aneurysmal subarachnoid hemorrhage and was found to have MMD. Also, we are conducting a systematic review to focus on the clinical association and prevalence of CNS vasculopathy in SS, and their presentations, management, and outcomes.

## Methods

### Literature review

#### Search methods

**Fig. 1:**
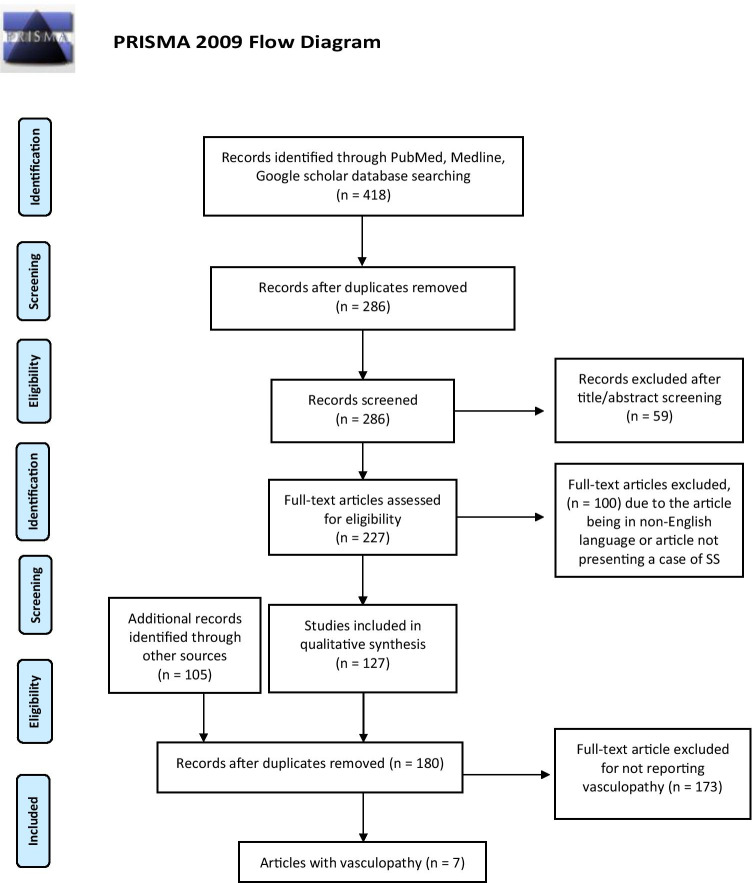
PRISMA diagram depicting the literature search strategy

We performed a comprehensive systematic review using the following keywords: “Seckel + syndrome” searching the MEDLINE, PubMed, Google Scholar, and Cochrane databases. It was conducted based on Preferred Reporting Items for Systematic Reviews and Meta-Analyses (PRISMA) guidelines. From the list of all reported cases, we looked for cases that reported CNS vasculopathies. To ensure that the list is comprehensive, we performed a comprehensive systematic review using the following keywords: “Seckel,” “Cerebrovascular disease,” “Vascular disorders,” “Aneurysms,” and “Moyamoya,” as MeSH and keywords in combination with Boolean operators to ensure inclusivity of all possible results. This too was conducted based on Preferred Reporting Items for Systematic Reviews and Meta-Analyses (PRISMA) guidelines. In addition, we identified candidate studies from the reference list of the eligible studies (Fig. 1).

#### Inclusion and exclusion criteria

SS was first reported in 1959; hence, all studies published from 1959 until May 15, 2021, which reported at least one case of SS, were included [8]. Only studies which were conducted in English or translated to English were included. Prenatal and autopsy diagnoses of SS as well as historical cases which were not confirmed were excluded. Furthermore, we assessed SS cases which reported CNS vasculopathies. Magnetic resonance angiography (MRA), digital subtraction angiography (DSA), cerebral angiogram (CTA), or autopsy findings were considered acceptable manners of diagnosing intracerebral vascular diseases. We considered digital subtraction angiography (DSA) as the diagnostic investigation for diagnosing MMD. However, the MRA and CTA findings provided by Inaloo et al. were considered acceptable to make the MMD diagnosis [128]. We have also assessed SS cases which reported CNS anomalies. The assessment was based on imaging of brain or autopsy findings of the brain if no imaging was performed.

### Selection of studies

Two of the reviewers (OK and SA) independently assessed the potential eligibility of each of the articles found through the database search. Determining the eligibility of the article was done through screening title/abstract then reviewing the full-text versions of the articles. All disagreements were resolved by a consensus, and by arbitration by a third reviewer (NA).

### Data collection

The data collected from each eligible article included the number of SS patients, the presence of a CNS anomaly, and the presence of a CNS vasculopathy. From articles which reported CNS vasculopathy, demographic data, clinical presentation, type of vasculopathy, Suzuki grading if the reported vasculopathy was MMD, treatment, surgical complications, and clinical outcomes were collected.

### Study objectives

The primary objectives of the study were (1) to review all the published literature pertaining to SS and (2) to review the cases of SS and vasculopathies by evaluating the clinical presentations, imaging results, treatment methods, and clinical outcomes.

The secondary objectives of the study were (1) to evaluate the proportions of CNS anomalies in SS, (2) to evaluate the proportions of vasculopathies and MMD in SS, and (3) to report a patient with SS and MMD.

### Quality assessment

Two reviewers (OK and SA) independently critically appraised the methodologic quality of the studies by using the modified tool suggested by Murad et al. [127]. This assessment aims to evaluate the risk of bias of the case reports and case series. We adapted this tool to assess the reported CNS vasculopathies in SS patients. Out of the 8 questions described in this tool, 5 questions were deemed compatible with our design and adjusted to fit our population. Each question can be answered with “Yes” or “No” after critically appraising each study. Studies were appraised based on the following: (1) if the study was specifically conducted to assess SS patients; (2) if the exposure of SS patients to treatment such as conservative treatment, pial synangiosis, endovascular treatment, or surgical clipping adequately ascertained; (3) if the outcome of SS patients with a vasculopathy, whether clinically or radiologically, is adequately ascertained; (4) if the SS patients were followed up for enough time for outcomes such as ischemic strokes, rebleeding, or re-rupture to occur; and (5) if the study was described with enough details for replication by another investigator or to allow other investigators to make an inference. A study was considered as high quality if it scored “Yes” in more than three questions, moderate quality if it scored “Yes” in two or three questions, and low quality if it scored “Yes” in one or none of the questions. All disagreements were resolved by a consensus, and by arbitration by a third reviewer (MB) (Supplementary table 1).

#### Ethical considerations

This study was approved by the Institutional Review Board at King Abdullah International Medical Research Centre (KAIMRC) with reference number: JED-21–427780-13450.

## Results

### Case illustration

This is an 8-year-old male patient, who is the second child of consanguineous parents, a product of preterm (26 weeks), and admitted to a neonatal intensive care unit (NICU) for 4 months. The patient, who is a known case of SS, presented to the emergency department with two episodes of tonic–clonic seizures, 20 min apart, and lasting for 10–15 min each, 2 h after a minor fall without losing consciousness, after which he resumed his activities.

**Fig. 2:**
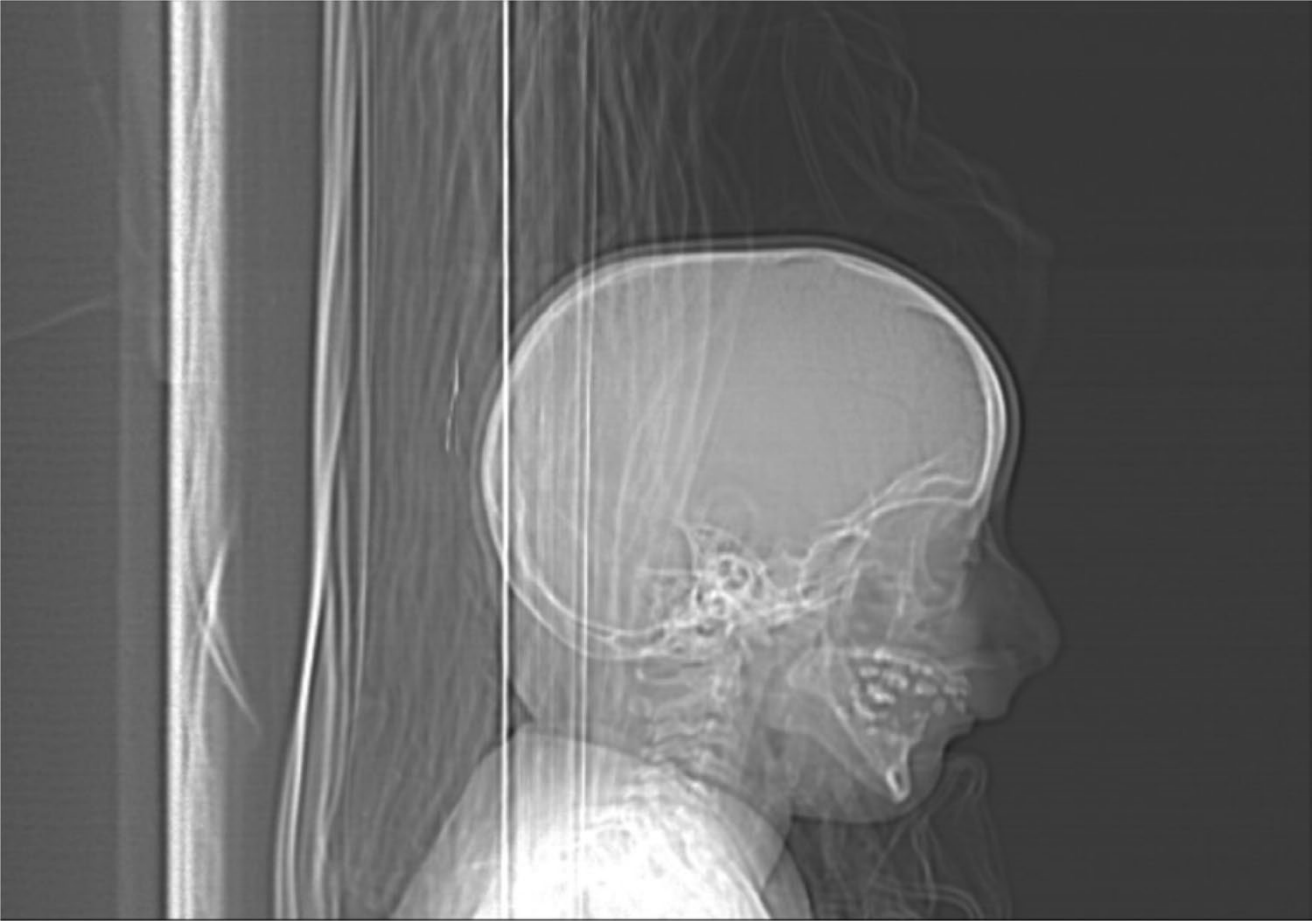
This is a scout image featuring the dysmorphic characteristics including micrognathia, beak-like nose, and receding forehead

**Fig. 3:**
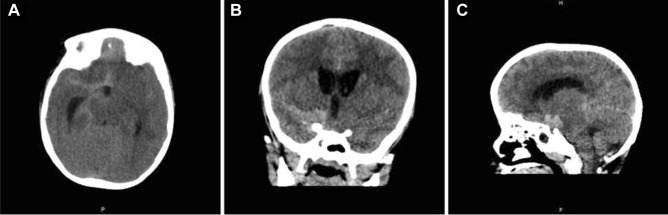
This is the initial computerized tomography (CT) scan with axial, coronal, and sagittal views demonstrating the basal subarachnoid hemorrhage (**A**–**C**)

**Fig. 4:**
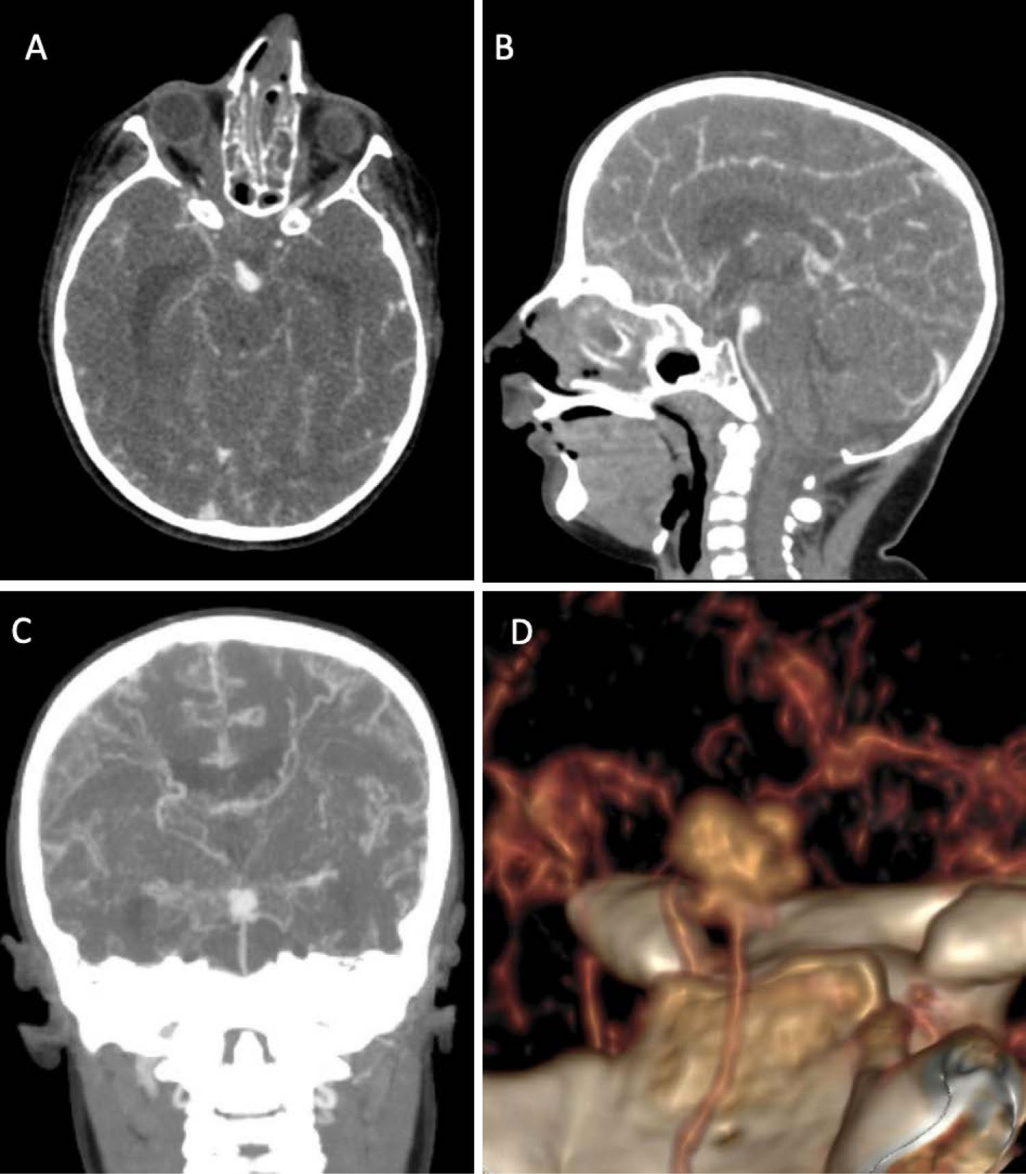
These are computerized tomography angiography (CT-A) axial, sagittal, and coronal images demonstrating the basilar tip aneurysm with an abnormal background of heavy collaterals (**A**–**C**); the basilar tip aneurysm has a multi-lobulated configuration illustrated in the three-dimensional image (**D**)

Upon examination, the patient was drowsy, and difficult to examine. He had a GCS of 11/15 (Eye 3/4, Verbal 3/5, Motor 5/6), pupils were 3 mm in size and reactive to light bilaterally, and he was able to move all limbs. Physical dysmorphic features were noted including microcephaly, microphthalmia, micrognathia, depressed nasal bridge, coloboma, and small hands and feet (Fig. 2). Urgent computed tomography (CT) of the brain showed evidence of significant subarachnoid hemorrhage (Fig. 3). The patient was admitted to the pediatric intensive care unit (PICU) for close observation. The patient underwent computed tomographic angiography (CTA) that demonstrated complete absence of flow in bilateral internal carotid arteries, as well as a basilar tip saccular aneurysm measuring 9 × 5 mm in maximum dimension (Fig. 4), and he was opted for endovascular management.

**Fig. 5:**
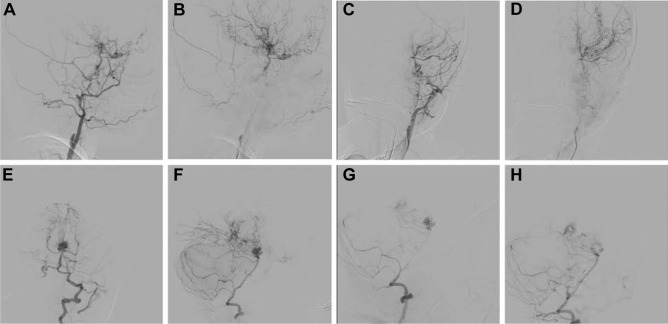
This is a digital subtraction angiography (DSA) lateral projection after right internal carotid injection showing complete occlusion with contrast reflux to right external carotid and its intracranial collaterals along with moyamoya features (**A**, **B**) with similar findings in the left side with posteroanterior (PA) views (**C**, **D**); right vertebral artery injection with ipsilateral oblique view in (**E**), and projection demonstrating the multi-lobulated basilar tip aneurysm before, during and after coiling (**F**–**H**)

**Fig. 6:**
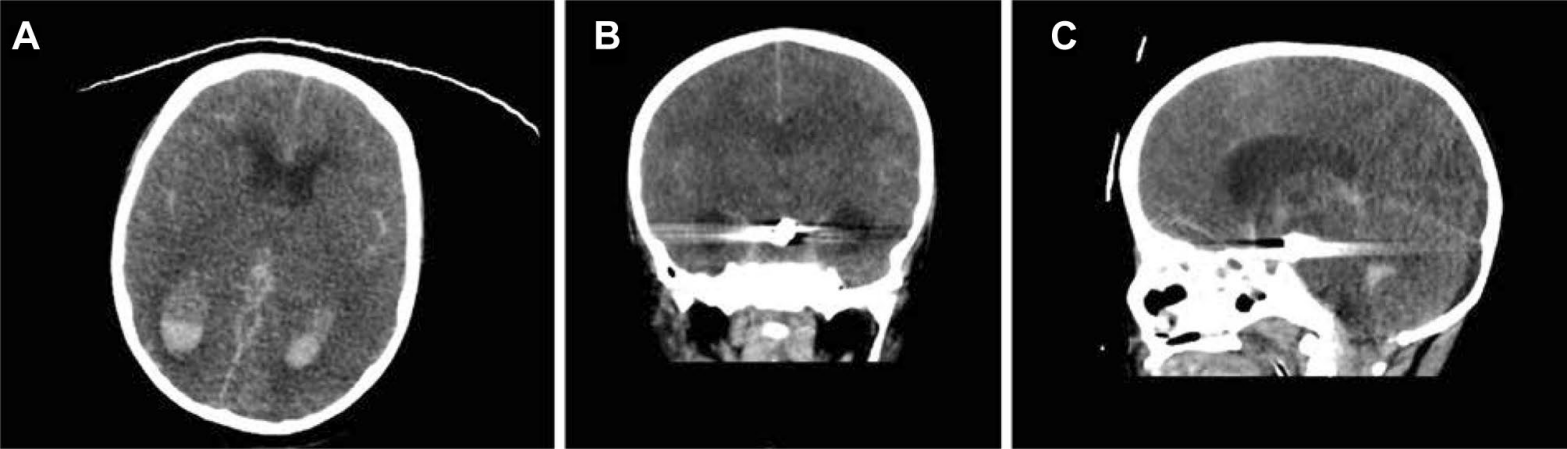
These are computerized tomography (CT) images after the sudden deterioration of the patient, showing intraventricular hemorrhage and further spread of the subarachnoid hemorrhage with hydrocephalus and significant edema (**A**–**C**)

On day 2, digital subtraction angiography (DSA) was done and a clear demonstration of MMD was visualized (Fig. 5). The patient underwent endovascular coiling of the basilar tip aneurysm with 9 coils leaving a minor filling residual of the sac; the procedure showed bilateral patency of the posterior cerebral arteries with no intraprocedural complications (Fig. 5). Shortly after returning to the PICU, the patient suddenly deteriorated, and urgent CT showed a significant interval increase in the subarachnoid hemorrhage with hydrocephalus associated with diffuse brain edema suggesting a rebleed of the aneurysm (Fig. 6). During the following day, despite maximal medical management and the patient was declared dead.

## Systematic review

After reviewing 418 articles, 286 articles were excluded as duplicates, and 59 of articles were excluded based on screening the titles or the abstracts. One hundred articles were excluded after reading full texts for not fulfilling the inclusion criteria. One hundred twenty-seven articles identified at least one SS case with the total number of SS patients being 252 patients [3–126]. Out of these articles, 7 reported a CNS vasculopathy in association with SS fulfilling the specified criteria (Fig. 1). 

Forty-eight articles reported CNS anomalies, of which 7 articles reported CNS vasculopathies (aneurysm and/or MMD). Including our case (n = 70/253), 27.67% of SS cases reported the presence of CNS anomalies including arterial aneurysms, vasculopathy, hematomas, clots, agenesis of corpus callosum, cysts, hypoplastic cerebrum or cerebellum, pachygyria, and herniations. The 7 articles reporting CNS vasculopathy in SS patients were extracted for further data analysis. After adding our case, the overall rate of CNS vasculopathy in SS patients is 3.16% (n = 8/253), where MMD accounted for 1.97% (n = 5/253). Aneurysms were mostly located in the basilar artery (50%), middle cerebral artery (37.5%), or the internal carotid artery (25%). Regardless of the management approach, 50% (n = 4/8) of the cases sustained mild-moderate neurological deficit, 37.5% (n = 3/8) died, and 12.5% (n = 1/8) sustained no deficit (Table 1) [3–7, 50, 127].

**Table 1:** Review of the reported SS cases with vasculopathy

Case number	Author/year	Age (Y)/gender	Presentation	Type of vasculopathy and its complication	Suzuki grading	Location of aneurysm	Treatment of MMD		Treatment of aneurysms		Perioperative complications	Duration of follow up	Outcome
							Conservative	Pial synangiosis	EVT	Clipping			
1	D’Angelo et al. 1998 [7]	17/F	Coma	Aneurysm, left intracerebral hematoma		B. MCA, B. ICA-PCoA, L. PCA-basilar tip, and ACoA complex				✔		6 months	Residual left hemiparesis
2	Sorof et al. 1999 [6]	19/M	Headache, cough, vomiting, rhinorrhea, and severe hypertension	Aneurysm, 30-mL right subdural clot (postmortem)		ICA-MCA bifurcation							Death
3	Di Bartolomeo et al. 2003 [4]	10/M	Convulsions, coma, and acute respiratory failure	Aneurysm, intraventricular hemorrhage with hydrocephalus		Basilar, L. PICA, and L. MCA			✔				Moderate dysphagia
4	Codd et al. 2009 [3]	16/F	Headache and numbness	MMD with aneurysm	III	L. PCoA		✔*	✔		Aneurysm rebleeding	1 year	Death
5	Rahme et al. 2010 [5]	18/F	Headache, generalized weakness, and worsening of right hemiparesis	MMD with aneurysm, spontaneous SAH with IVH		PCA	✔						No deficits
6	Inaloo et al. 2016 [128]	6/F	Sudden weakness, left facial drooping, and inability to walk	MMD	I-II			✔				4 months	Ambulatory with mild weakness
7	Gunesli et al. 2018 [50]	12/M	Convulsions	MMD with aneurysm, left subdural hematoma		L. MCA, R. ICA, and basilar tip			✔		Ophthalmoplegia	9 months	Ophthalmoplegia
8	Current study, 2021	8/M	Convulsions	MMD with aneurysmal SAH	IV	Basilar tip			✔		Aneurysm rebleeding, and hydrocephalus		Death

*MMD* moyamoya disease, *SAH* subarachnoid hemorrhage, *IVH* intraventricular hemorrhage, *ACoA* anterior communicating artery, *MCA* middle cerebral artery, *ICA* internal carotid artery, *PCA* posterior cerebral artery, *PCoA* posterior communicating artery, *PICA* posterior inferior cerebellar artery, *EVT* endovascular treatment, *L* left, *R* right, *B* bilateral

^*^Pial synangiosis was performed 1 year prior to the endovascular treatment, after which the patient remained asymptomatic for 12 months

### Literature review

#### Demographic data and clinical presentations

A total of 8 cases with SS have been identified in literature that is associated with CNS vasculopathy, including our illustrative case (Table 1). The mean age of diagnosis was 13.25 (range 6–19) years with equal gender distribution (M:F, 1:1). Most common symptoms/presentations were headache (37.5%), seizure (37.5%), followed by weakness (25%), or coma (25%). All but one patient (87.5%) were found to have cerebral aneurysms either isolated or with a background of MMD. MMD was diagnosed in 62.5% of the patients. All patients were diagnosed due to complications of their disease rather than being diagnosed as a part of screening.

Codd et al. reported the first case in 2009 which draws the attention toward the possible association between SS and MMD. Of the cases reported to have both SS and MMD, three presented with signs of cerebral ischemia including unilateral weakness, numbness, facial drooping, and inability to walk, whereas 1 case presented with convulsions [3, 5, 50, 128].

#### Vasculopathy characteristics

All SS patients with MMD had bilateral pathology with no predilection to a specific severity grade. Suzuki grading of MMD in SS patients was reported in 2 cases, Codd et al. reported a Suzuki stage of III, and Inaloo et al. reported a mild stage of I–II. In our case, conventional subtraction angiography confirmed the diagnosis with a Suzuki grade of IV. Majority (71.4%) of SS with aneurysms had posterior circulation involvement [128].

#### Management and outcomes

A single case of SS with aneurysm, reported by D’Angelo et al. was managed with clipping. The remaining patients in this group were managed either with endovascular control or conservatively. Three patients (37.5%) of SS with CNS vasculopathy died from disease, two of which were due to aneurysmal rebleeding following endovascular intervention [3–7, 50, 128].

## Discussion

SS was first reported in 1959 by Mann and Russell and later described in detail by Seckel in 1960. It is characterized by short stature, microcephaly, moderate-to-severe mental retardation, cryptorchidism, and facial abnormalities [8, 130]. Dysmorphic features include, but are not limited to, a receding forehead, narrow face, large eyes, large beak-like protrusion of the nose, and micrognathia. On top of these distinct physical features, our case was confirmed to have SS via molecular testing and presence of PCNT mutation. PCNT gene mutations are typically indicative of MOPD II; however, it has been reported to be present in SS patients as well. Willems et al. noticed that SS patients who have PCNT mutation seem to have more severe growth retardation than those who do not have the gene mutation (−6 to −8 SD vs. −4 to −5 SD) [131]. It is important to note that MOPD II is frequently misdiagnosed as SS due to the similarities between them. Moreover, correctly diagnosing SS becomes especially difficult when the patient does not present with all of the features of SS [132]. One way to differentiate is to examine the proportionality as SS exhibits proportionate growth retardation and MOPD II exhibits disproportionate growth retardation [66].

MMD is a rare chronic occlusive cerebrovascular disease which is characterized by unilateral or bilateral progressive narrowing of the circle of Willis as well as abnormal system of blood vessels at the base of the brain [129, 133]. Suzuki and Takaku were the first to describe MMD in 1969 as small, basal, and collateral vessel formation in a patient with bilateral occlusion of the internal carotid arteries which showed the appearance of a puff of smoke on imaging [134]. MMD seems to have a bimodal distribution with the first peak occurring around the age of 10 years and the other around the age of 30–40 years. Although this syndrome has a higher incidence in individuals of Asian descent, it is increasingly recognized throughout the world, with a prevalence of 3 cases per 100,000 children in Japan and 0.086 cases per 100,000 persons in America [133, 134]. In comparison to the number of MMD cases in SS that we estimated, the number of MMD in these populations is drastically lower. MMD has been linked to other conditions like atherosclerosis, autoimmune diseases, meningitis, sickle cell anemia, and trisomy 21. Also, MMD patients generally have a higher incidence of aneurysm formation in the posterior cerebral circulation [3, 127, 135]. The gold standard for diagnosis and evaluation of MMD is digital subtraction angiography [50].

Particularly in pediatrics and patients with MPDs, like SS, cerebral ischemia is the most often encountered clinical presentation of MMD [3, 128, 140]. The etiology in our patient was an aneurysmal subarachnoid hemorrhage (SAH) from the basilar tip aneurysm that caused convulsions, on a background of advanced MMD. Posterior circulation aneurysm formation in MMD is a reported risk due to the high flow pressure shifted especially at the basilar tip, and this is clearly demonstrated in SS patients (Table 1). Pediatric cerebral aneurysms are a relatively uncommon encounter in clinical practice as reports suggest an incidence between 3 and 4%, which translates to roughly 1–3 cases per 1 million population [136]. The majority of cases present within the first 2 years of life with a peak in the first 6 months after birth [136]. These early childhood lesions typically arise in the vertebrobasilar system and along the middle cerebral artery [136, 137]. The presence of multiple aneurysms in this age group is rare, accounts for around 5% of cases, and is typically associated with other factors such as prior cranial irradiation, MMD, sickle cell disease, and arteriovenous malformations [136]. SAH is the most common initial presentation for cerebral aneurysms [136, 138]. Medical management should be initiated immediately to prevent secondary complications such as rebleeding and to restore hemodynamic stability [136]. Surgical options include aneurysm neck occlusion or ligation, occlusion of the carrying artery of aneurysm, aneurysm wrapping with muscle, aneurysm resection, or carotid artery ligation [137]. Both surgical and endovascular management showed good outcomes; however, endovascular management requires close monitoring to detect any aneurysmal propagation especially with such long-life expectancy [137].

Numerous reports have indicated the association between various phenotypes of microcephalic dwarfism and cerebrovascular lesions, both occlusive and aneurysmal [139, 140]. A 2013 review of all reported cases of MOPD II to establish an evidence-based screening approach found that the frequency of cerebrovascular lesions in this subset of patients to be approximately 44% (95% CI 19–52%), not far from an earlier reported frequency of 52% [139, 140]. Additionally, younger children tend to present with occlusive arteriopathy while older patients have aneurysms [3, 139, 141]. A proposed recommended screening approach is to initiate screening at time of diagnosis using magnetic resonance imaging (MRI) and magnetic resonance angiography (MRA) of the circle of Willis and cervical arteries. MRA is preferred for screening as it spares patients from the risk of intracranial hemorrhage and adverse consequences of more invasive modalities [139, 140]. Considering the uncertainty about the development and progression of cerebrovascular lesions in this population, screening should be performed early and frequently. Imaging should be on a yearly interval up to 10 years of age and thereafter widened to every 2 years with the goal of detecting aneurysms. Current data suggest this approach to be lifelong. This tactic is supported by a large dataset, high rate of cerebrovascular anomalies among this population, adverse outcomes if left untreated, and the potential of early detection and prophylactic treatment to improve long-term outcomes [139, 140]. We believe that such a screening should also be applied to patients with SS, giving the relative similarities between both syndromes, and the recent appreciation of increased rate of neurovascular anomalies is SS compared to the normal population. We agree with Perry et al., and we favor the use of MRA as a screening tool as it spares patients from the risk of contrast, radiation associated with DSA and CTA, and adverse consequences of more invasive modalities. This is also supported by a recent systematic review paper discussing the intracranial aneurysms in all MPD patients, inclusive of SS [142].

Management of MMD is aimed to improve perfusion of the affected region of the brain. This improvement has the potential of reducing risk of future stroke as well as mitigating symptoms [3, 127]. Conservative medical treatment is used when the patient is deemed high risk for invasive procedures or in case the condition is mild [143]. Since the pathology of MMD spares the external carotid artery, it is typically used as a source of new blood flow either directly or indirectly [3, 143]. Direct revascularization involves the anastomoses of a branch of the external carotid directly to a cortical artery. On the other hand, indirect revascularization is achieved by promoting new vessel formation by placing a tissue perfused by the external carotid in direct contact with the brain [135, 143]. A review of 143 children who underwent pial synangiosis demonstrated that perioperative stroke frequency was reduced from 67% down to 3.2% after ≥ 1 year postoperatively [135, 143]. Open surgical revascularization is technically difficult in children given the small size of anastomosed vessels. This is clearly further aggravated in cases of microcephalic dwarfism [143, 144]. Additionally, patients with microcephalic dwarfism should be treated akin to infantile patients as their cerebral autoregulation is underdeveloped [129]. The paucity of evidence on the optimum management approach along with the associated comorbidities that impact outcomes in these patients warrants a case-by-case approach to management. Additional studies are needed to delineate the difference in outcomes, or lack thereof, between different treatment modalities.

Our review for the management of MMD associated with SS was managed conservatively only once and was managed by indirect revascularization twice. Both cases managed surgically had favorable outcomes as one was treated with encephalomyosynangiosis and demonstrated reasonable recovery after 4 months with residual weakness in right lower extremity while the other was managed with pial synangiosis and had reasonable outcome at 1-year follow-up (asymptomatic, no headache, or transient ischemic attack (TIA)) [3, 5, 50, 127]. This data, although small in number, carries support to manage MMD cases surgically early in the course in order to reduce the morbidity. Prevention of aneurysm formation by this intervention is a valid question that requires large prospective studies that are still lacking. However, a 10-year prospective study for patients with aneurysms coexistent with MMD reported benefit from surgical revascularization to indirectly treat peripherally located aneurysms [145].

In the case of SS-associated aneurysms, our review included a single case with surgical clipping that resulted in relatively good recovery and aneurysm control. The other four cases (including our case) were managed endovascularly with acceptable outcomes in 2 cases (50%), and rebleeding causing death in the remaining two cases (50%). There were a total of three cases of SS-associated aneurysms with a background of confirmed MMD and were all managed endovascularly with 2 out of 3 cases resulting in a rebleeding and death (Table 1). With such small number of cases, a predilection of the appropriate treatment methodology is difficult to be drawn, although endovascular intervention for aneurysms in MMD in this review reflected discouraging results. Case-by-case decision making seems most appropriate until further pooling of data is reported with this context of co-existing pathologies.

### Limitations

Considering the retrospective nature of reports included in this review, publication bias is an unavoidable limitation to this review. The inconsistent reporting of several variables such as aneurysm morphology, treatment plan, and outcomes may also hinder the ability to make conclusions regarding the optimum management of this patient population. A key limitation to this review was the language barrier that impeded the authors’ capacity to evaluate reports written in languages other than English, of which there were many. Republication in which the same case was reported multiple times may have introduced bias as well. Epidemiological data are difficult to be concluded for such a rare entity; we understand that using cohort studies is the best way to infer these statistics. However, in our case, we tried to pool all the cases of SS and report a representative estimate of the prevalence for vasculopathies in SS. Nevertheless, it is our belief that this review provides an outline that could inspire additional exploration of the association between SS and CNS vasculopathy.

## Conclusion

High index of suspicion should be maintained in SS patients, especially when presenting with focal neurological deficits, and MMD should be part of the differential diagnosis. Prevalence of CNS vasculopathy in SS is 3.16% with a much higher prevalence of MMD compared to the general population. Screening for cerebral vasculopathy in SS is justifiable especially in centers that have good resources. Further data are still needed to identify the most appropriate management plan in these cases.

## Supplementary Information

Below is the link to the electronic supplementary material.

Supplementary file1 (PDF 67 KB)
