# Supplementary material for: Central nervous system vasculopathy and Seckel syndrome: case illustration and systematic review
Source: Childs Nerv Syst. 2021 Aug 3;37(12):3847–60. doi: 10.1007/s00381-021-05284-8 (PMC8604825; doi:10.1007/s00381-021-05284-8)
Supplement: Supplementary file 1 — Supplementary file1 (PDF 67 KB) [file 381_2021_5284_MOESM1_ESM.pdf]

Supplementary table 1: Quality assessment of Seckel syndrome studies with a vasculopathy

**Study**	**Does the patient(s) represent(s) the whole experience of the investigator in the study (was the series specifically related to Seckel syndrome)?**	**Was the exposure (e.g conservative, pial synangiosis, endovascular treatment, surgical clipping) adequately ascertained?**	**Was the outcome (clinically and radiologically) adequately ascertained?**	**Was follow-up long enough for outcomes (ischemic stroke, aneurysm recurrence, or re-rupture) to occur?**	**Is the case(s) described with sufficient details to allow other investigators to replicate the research or to allow practitioners make inferences related to their own practice?**	**Quality**
D'Angelo et al, 1998 [7]	Yes	Yes	Yes	Yes	Yes	High
Sorof et al, 1999 [8]	Yes	No	No	Yes	No	Moderate
Di Bartolomeo et al, 2003 [9]	Yes	No	No	No	No	Low
Codd et al, 2009 [4]	Yes	Yes	Yes	Yes	Yes	High
Rahme et al, 2010 [10]	Yes	No	No	No	Yes	Moderate
Inaloo et al, 2016 [11]	Yes	Yes	Yes	Yes	Yes	High
Gunesli et al, 2018 [12]	Yes	Yes	Yes	Yes	Yes	High

* Reports with at more than three positive quality assessment criteria were considered low risk of bias, two or three positive qualities as medium risk, and one or none positive qualities was considered as high risk or bias.
